# Transplantation of iPSC-derived vascular wall-like tissue for improved embolization and reconstruction of aneurysm sac in endovascular aneurysm repair

**DOI:** 10.3389/fbioe.2026.1865298

**Published:** 2026-09-21

**Authors:** Zhi-Yong Li, Jie-Fu Fan, Xiao-Nan Wang, Yang-Kai Wang, Hao Zhang, Biao Wu, Lei Zhang

**Affiliations:** 1 Department of Pathology, Faculty of Medical Imaging, Naval Medical University Shanghai/Second Military Medical University, Shanghai, China; 2 Department of Vascular Surgery, Changhai Hospital, Navy Medical University/Second Military Medical University, Shanghai, China; 3 Naval Medical Center of PLA, Shanghai, China

**Keywords:** aortic aneurysm, cell therapy, endoleak, endovascular aneurysm repair, induced pluripotent stem

## Abstract

**Objective:**

Endoleaks are major complications of endovascular aneurysm repair (EVAR) for treating abdominal aortic aneurysms. Sac embolization is a crucial therapeutic method, in which cell transplantation offers advantages. To attain improved survivability and adaptability, vascular wall-like tissues derived from human induced pluripotent stem cells (iPSCs) were fabricated, and the feasibility of their transplantation for the treatment of endoleaks following EVAR was investigated.

**Methods:**

Human iPSCs were cultured in suspension to generate embryoid bodies, which were subsequently differentiated into smooth muscle cell-lineage-associated cells. Cell aggregates were embedded within Matrigel and cultured to form vascular wall-like tissues (VWTs). VWTs were subsequently transplanted into a rat model of aneurysm with endoleak. Histological analyses, including hematoxylin and eosin staining, Masson’s trichrome staining, and immunofluorescence, were performed to evaluate the embolization effects and tissue regeneration in the aneurysm sac.

**Results:**

Our study successfully established VWTs and a novel rat aneurysm model with endoleak. The transplantation of VWTs resulted in near-complete histological filling of the aneurysm sac over a period of 4 weeks. During this time, the proportions of both solid filling area and nucleated cells increased, while the proportion of acellular structure correspondingly decreased. Intriguingly, the regenerated tissue was predominantly composed of α-SMA^+^ cells, originating from both the transplanted tissue and host cells.

**Conclusion:**

Transplantation of VWTs into the aneurysm sac elicited favorable embolization effects and facilitated tissue regeneration and structural reconstruction of the aneurysm sac, which holds potential for long-term embolization of the aneurysm sac in EVAR.

## Introduction

Endovascular aneurysm repair (EVAR) has become a crucial treatment for abdominal aortic aneurysms (AAA), favored over open surgery due to its minimally invasive nature (Isselbacher et al., 2022). However, endoleak, a major complication of EVAR, arises from incomplete aneurysm sac exclusion and persistent blood flow. This diminishes the overall efficacy of EVAR and increases the need for re-interventions (Schmitz-Rixen et al., 2020; Wu et al., 2024). Addressing endoleaks is thus critical to optimizing EVAR efficacy.

Sac embolization and filling have emerged as highly effective methods for preventing and treating endoleaks in EVAR (Marchiori et al., 2022; Nana et al., 2022). During EVAR, surgeons can deliver embolic materials directly into the aneurysm sac through a pre-positioned catheter after deploying the stent graft (Isselbacher et al., 2022). This technique effectively seals the sac, preventing persistent pressurization from endoleaks. Evidence suggests that sac embolization promotes thrombus formation, reduces backflow, enhances sac regression, and decreases the need for re-interventions (Schmitz-Rixen et al., 2020; Wu et al., 2024). Notably, it significantly reduces Type II endoleak incidence and late sac expansion rates, potentially improving long-term EVAR durability (Marchiori et al., 2022; Nana et al., 2022). Various embolic agents, such as thrombin, glue, coils, and hydrogel, have been developed to fill and seal the aneurysm sac, aiming to eliminate the residual sac and the risk of endoleak (Marchiori et al., 2022; Nana et al., 2022; Faries et al., 2003; Zhang et al., 2019; Zehtabi et al., 2017). While these agents yield favorable immediate results, their long-term efficacy is limited by degradation, deformation, and displacement within the sac (Nana et al., 2022).

Mechanistically, current sac embolization therapies rely on mechanical sealing with artificial agents, which may impede natural healing mechanisms and aortic wall reconstruction (Hinchliffe, 2017; Hu et al., 2019). Therefore, an ideal embolic agent should not only achieve immediate aneurysm exclusion but also actively promote healing and tissue regeneration within the residual sac (Hu et al., 2019).

Cell therapy, particularly stem cell transplantation, holds remarkable potential for the structural and functional restoration of arterial walls (Adibi et al., 2016; Bashur et al., 2013). Introducing cells into the aneurysm sac can expedite thrombosis organization, collagen synthesis, and tissue plasticity preservation (Adibi et al., 2016). Induced pluripotent stem cells (iPSCs) can be derived directly from a wide range of adult cell types and offer distinctive advantages due to their paracrine effects, high proliferation rates, and may offer immunologic advantages (Bashur et al., 2013; Xie et al., 2011; Marbacher et al., 2014). Thus, cell therapy could not only fill the aneurysm sac directly but also potentially trigger local regenerative mechanisms, thereby enhancing treatment efficacy regarding occlusion, durability, and safety. However, ongoing studies focusing on cell suspension injections for aneurysm embolization have produced mixed results (Adibi et al., 2016; Marbacher et al., 2014; Kajimoto et al., 2006), possibly due to the hypoxic and nutrient-deficient environment of the aneurysm sac, which could negatively affect cell survival and proliferation.

Three-dimensional engineered tissue constructs, including stem cell–derived tissue sheets and cell clusters, offer advantages over dissociated cell suspensions by preserving cell–cell interactions and a local microenvironment, thereby improving post-transplantation viability, host integration, and paracrine activity (Hu et al., 2023; Nakao et al., 2023; Zhang et al., 2024; Kajita et al., 2024). Notably, iPSC-derived retinal sheets and engineered cardiac tissues have shown superior survival compared with injection of cell suspensions (Watari et al., 2023; Akbarzadeh et al., 2023). However, the potential of such engineered cell clusters for aortic aneurysm sac embolization has not been studied, and further work is needed to clarify their suitability for improving aneurysm embolization and endoleak treatment outcomes.

In the present study, we cultured and differentiated iPSCs *in vitro* to fabricate vascular wall-like tissues (VWTs) and evaluated their application in an aneurysm-endoleak model. We hypothesized that transplantation of VWTs into the aneurysm sac could achieve durable embolization and promote tissue regeneration and sac remodeling, thereby providing a feasible strategy for treating endoleaks after EVAR.

## Methods

### Preparation and development of VWTs

Induced pluripotent stem cells (iPSCs) were chosen for the construction of VWTs due to several key advantages: their ease of acquisition through the reprogramming of fibroblasts, robust proliferative capacity and differentiation potential, and reduced ethical concerns (Yan et al., 2017). Human iPSCs (DYR0100, SIBS Cell Bank, China) were cultured on Matrigel-coated dishes (Xiamen Moji Biotech, China) in complete mTeSR™1 medium (STEMCELL Technologies, Canada). A ROCK inhibitor, Y27632 (10 μM, Abmole), was added to the medium to enhance cell survival and prevent apoptosis during the initial stages of culture until the cells reached 80% confluence. Subsequently, the cells were transferred to an ultra-low attachment surface plate (STEMCELL Technologies, Canada), a standard approach for tissue culture that mimics *in vivo* organ development, to facilitate the formation of embryoid bodies (EBs), three-dimensional cell aggregates.

After two days, the medium was replaced with an EB-mesodermal differentiation medium, consisting of complete mTeSR™1 medium supplemented with CHIR99021 (10 μM, Abmole) and recombinant protein BMP4 (30 ng/mL, Abmole). These factors induce mesodermal specification.

Two days later, the medium was switched to a Mesodermal-smooth muscle cell (SMC) differentiation medium, enriched with PDGF-B (10 ng/mL, Abmole) and TGFβ1 (2 ng/mL, Abmole). These growth factors promote the differentiation of mesodermal cells into SMC-lineage-associated cells, the primary cellular components of the vascular wall. This medium was refreshed every two days for one week to ensure continuous exposure to differentiation signals.

After seven days, the EBs were collected and sandwiched between two layers of Matrigel for further culture in the Mesodermal-SMC differentiation medium over seven weeks. This process folds the three-dimensional EBs into a sheet-like form, facilitating delivery through a 5F catheter and syringe needle with a small diameter, as required for clinical use. After seven weeks of culture, VWTs were harvested under sterile conditions. Pre-cooled Cell Recovery Solution (Corning, United States) was added to Matrigel-embedded VWTs and incubated at 4 °C until the matrix was loosened. VWTs were collected by centrifugation at 300 g for 5 min, resuspended in sterile PBS, and immediately injected into the aneurysm sac through a 5F catheter or small-gauge needle.

The differentiated cells were characterized using immunofluorescence staining for the smooth muscle cell marker, smooth muscle alpha-actin (α-SMA) (BM0002, BOSTER). Additionally, human-specific anti-lamin A antibody (ab215324, abcam) was employed to distinguish human cells.

### Animals and general procedures

Sixteen-week-old male Sprague Dawley (SD) rats (weight: 260–300 g) were supplied by Beijing Vital River Laboratory Animal Technology Co., Ltd (Beijing, China). Rats were housed in a temperature-controlled room and kept on a 12:12 h light-dark cycle with free access to food and water. All procedures in this study were approved by the Ethical Committee. All operations were conducted following the Guide for the Care and Use of Laboratory Animals published by the United States National Institutes of Health (NIH publication No. 85–23, revised 1985). The work has been reported in accordance with the ARRIVE guidelines. At the predetermined endpoints, rats were euthanized by an overdose of anesthetic before tissue harvest.

Five experimental groups were established: (1) a sham group, which underwent identical surgical exposure and vessel manipulation (ligation and incision) but without mechanical dilation or fluid injection; (2) a normal saline (NS) group, in which the aneurysm sac was injected with normal saline as a negative control; (3) a Matrigel group, in which the sac received Matrigel alone (without VWTs) as a vehicle control; (4) a VWT group, in which the sac was injected with VWTs suspended in Matrigel; and (5) a model group of untreated aneurysms used solely for model characterization. For each group, five rats were allocated to each of three time points (2, 3, and 4 weeks post-surgery), giving a total of 75 animals (5 groups × 3 time points × 5 rats per time point). Aneurysm tissues from every animal were processed for both histological staining and immunofluorescence using adjacent serial sections. Accordingly, all quantitative data reported for a given group at a given time point represent five biological replicates (n = 5).

An additional assessment of CsA efficacy included vehicle (aneurysm modeling and NS filling without CsA), CsA (aneurysm modeling and NS filling with CsA), and CsA + VWTs (aneurysm modeling and VWT filling with CsA) groups (n = 5 per group).

### Establishment of an aneurysm model with endoleak

Rats were fixed on a surgical board (Alcott Biotech, Shanghai) with continual 3% inhaled isoflurane for gaseous anesthesia. As shown in Figure 1, the common iliac artery, external iliac artery, and internal iliac artery were surgically dissected and exposed. The distal end of the external iliac artery and the proximal end of the common iliac artery were ligated, and the internal iliac artery was transiently occluded using a mosquito clamp. A minor incision was created at the common iliac artery. A syringe was introduced into the common iliac artery, and normal saline (NS) was infused with gradually increasing pressure. When the arterial diameter reached 1.5–2 times its original size, the pressure was maintained at that level to ensure consistent expansion across animals within the same batch. The fluid from the artificial aneurysm was extracted. Subsequently, according to group assignment, the aneurysm sac was injected with either normal saline (NS group), Matrigel alone (Matrigel group), or VWTs suspended in Matrigel (VWT group), followed by prompt ligation of the incision. In the sham group, the incision was ligated without any injection. After 5 minutes, the clamp was removed, allowing reperfusion from the internal iliac artery to create an endoleak model. After surgery, each rat received intraperitoneal injections of cyclosporine A (CsA) at a dosage of 10 mg/kg/day for four weeks to prevent immune rejection, and intramuscular injections of penicillin G at a dosage of 50 mg/kg/day for 1 week to prevent infection.

**FIGURE 1:**
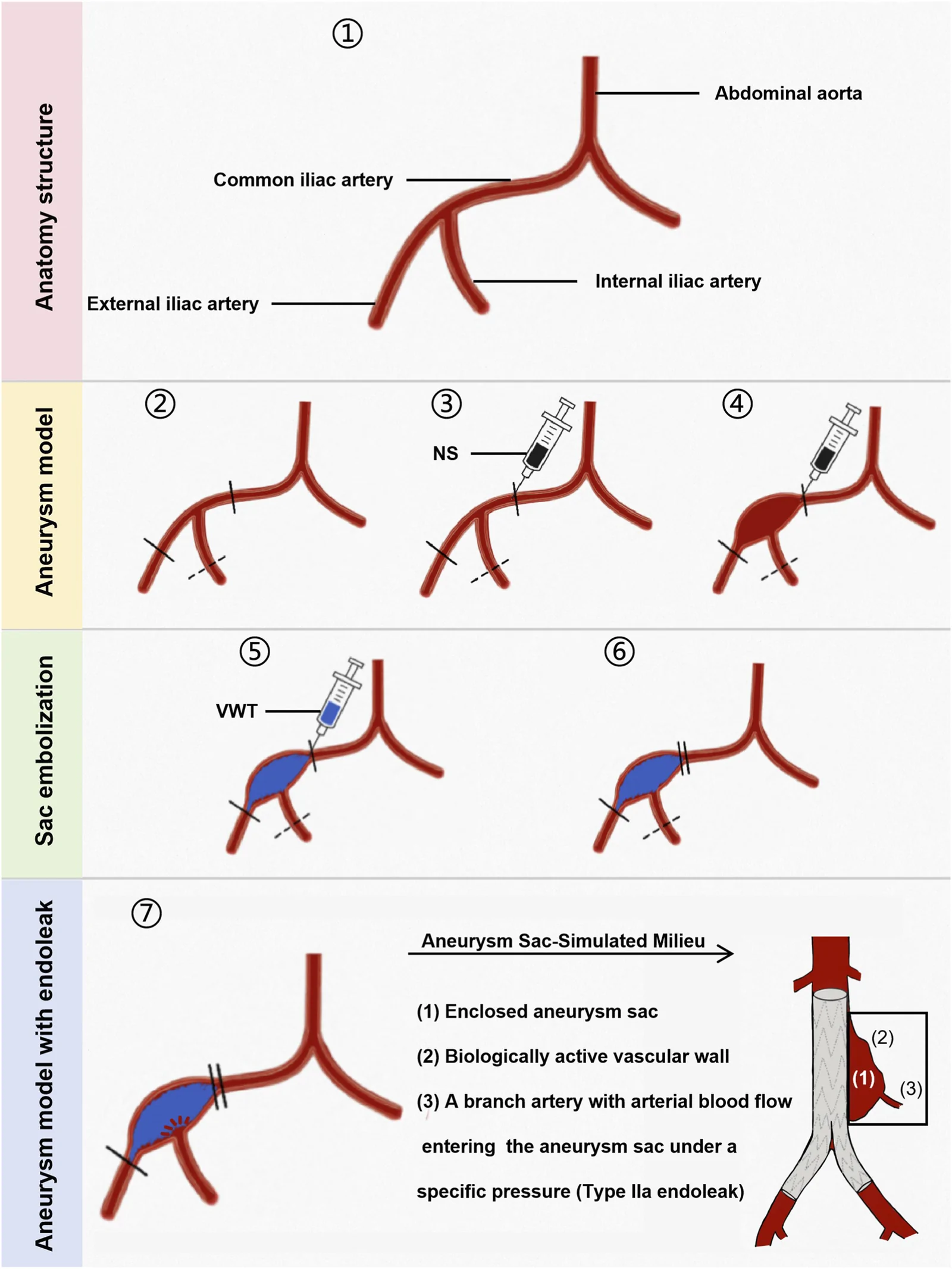
Establishment of an aneurysm model with endoleak. (1) The common iliac artery, external iliac artery, and internal iliac artery were surgically exposed; (2) The distal end of the external iliac artery and the proximal end of the common iliac artery were ligated, and the internal iliac artery was transiently occluded; (3) A syringe was introduced into the common iliac artery; (4) Normal saline (NS) was infused while maintaining constant pressure; (5) vascular wall-like tissues (VWTs) were injected into the aneurysm sac; (6) Prompt ligation of the incision; (7) After 5 min, the clamp was removed, allowing reperfusion from the internal iliac artery to create an endoleak model.

To confirm the presence of endoleak, after release of the clamp and restoration of retrograde perfusion, the aneurysm sac was cannulated and the intra-aneurysm blood pressure (BP) and heart rate (HR) were recorded by the PowerLab system (AD Instruments, Australia) for at least 10 min.

### Histological analysis

Arterial tissues were harvested in the modeling location. At the time of tissue harvest, the aneurysm sac and its surrounding surface were gently rinsed with physiological saline to remove uncoagulated fresh blood, thereby avoiding interference with subsequent histological interpretation. The tissues were then fixed in 4% paraformaldehyde, dehydrated, and embedded in paraffin to prepare paraffin blocks. Hematoxylin and eosin (H&E) staining, Masson’s trichrome staining, and Elastic Verhoeff-Van Gieson (EVG) staining were performed on 5 μm sections as previously described (Yin et al., 2024). Briefly, the sections were stained using the H&E Staining Kit (Solarbio, Beijing, China) and Masson’s Trichrome Stain Kit (Solarbio). For EVG staining, the sections were immersed in EVG solution for 30 min, followed by incubation with ferric chloride differentiation solution until the background was grey white. Then, the sections were re-stained with VG solution. After dehydration using 100% ethanol, the sections were photographed under a light microscope. The histological results were observed and imaged using a microscope of Nikon (ECLIPSE CI, Japan).

On H&E-stained sections, the solid embolization area was defined as the region filled with tissue blocks and Matrigel within the aneurysm sac, while the total embolization area included both solid filling and blood clot filling. Both areas were outlined manually and measured using ImageJ, and expressed as percentages of the total sac area.

As previously reported (Yin et al., 2024), the middle layer structure of the artery in EVG staining sections was observed. Elastin fragmentation grades were evaluated based on the degree of elastin fiber rupture and the integrity of the media lamellar units as follows: Grade 1: No significant rupture of elastin fibers, with intact elastin lamellae. Grade 2: Partial rupture of elastin fibers, with the lamellar structure still largely intact. Grade 3: Severe rupture of elastin fibers, with interlacing and disorder in the lamellar layers. Grade 4: Disordered arrangement of elastin fibers, with the lamellar hierarchy no longer recognizable. Grades 2–4 are considered positive for elastin fiber and lamellar disruption.

Masson’s trichrome-stained sections were used to quantify collagen deposition within the aneurysm sac. Collagen fibers appeared blue, while muscle and elastic fibers appeared red. Images were converted to grayscale, and the blue-stained collagen area was identified by color threshold segmentation. The collagen area ratio was calculated as the percentage of collagen-positive area relative to the total aneurysm sac area.

### Immunofluorescence

The harvested tissue sections were deparaffinized with xylene and rehydrated through a graded ethanol series. Antigen retrieval was performed by microwave heating. The sections were incubated with 3% hydrogen peroxide at room temperature for 15 min, followed by blocking with 10% goat serum for 15 min. Triple staining was performed using a multiplex immunofluorescence staining kit (AFIHC027, AiFang Biological, China). In each round, the sections were incubated overnight at 4 °C with a single primary antibody against α-SMA (1:1000, BM0002, BOSTER), CD31 (1:1000, A3684, R&D), or vimentin (1:1,000, 10366-1-AP, Proteintech), followed by three washes with PBST. Polymer-HRP anti-mouse/rabbit universal secondary antibody IgG (AFIHC001, AiFang Biological) was applied for 30 min at room temperature. After washing, tyramide (TSA) dyes were added and reacted for 8 min. The sections were washed with PBST, and the antibodies were stripped by microwave treatment. This cycle was repeated to complete the labeling of all targets. Nuclei were counterstained with DAPI for 10 min at room temperature in the dark. Sections were mounted with an anti-fluorescence quenching mounting medium and imaged using an eight-channel fluorescence digital slide scanner (AF-KL-20–8, AiFang Biological).

For cell quantification, α-SMA^+^/vimentin^−^ cells, vimentin^+^/α-SMA^-^ cells, and CD31^+^ cells were quantified as three marker-defined cell populations. The α-SMA^+^/vimentin^−^ population was operationally considered SMC-lineage-associated in this staining scheme. Total cell numbers were determined by DAPI staining, and the percentage of each marker-defined population was calculated accordingly. Nucleated cell count was expressed as fold change relative to the 2-week time point. All quantifications were performed by two independent observers blinded to the experimental groups.

### Quantitative real-time PCR analysis

Total RNA was extracted from peripheral vascular wall tissues using TRIzol reagent (Invitrogen, United States) according to the manufacturer’s protocol and reverse-transcribed into cDNA using the PrimeScript RT reagent kit (Takara, Japan) with 0.5 μg of RNA as template. For detection of human graft-derived cells in rat tissues (biodistribution analysis), genomic DNA was extracted from the aneurysm sac and various organs (brain, lung, heart, liver, kidney, and spleen) using the DNeasy Blood & Tissue Kit (Qiagen, Germany) according to the manufacturer’s instructions. Quantitative real-time PCR (qPCR) was performed on a CFX-96 real-time PCR detection system (Bio-Rad, United States) using the SYBR Green kit (Takara, Japan). For rat cytokine mRNA expression, the following rat-specific primer pairs were used: for Gapdh (internal control), forward 5′-CTG​GAG​AAA​CCT​GCC​AAG​TAT​G-3′ and reverse 5′-GGT​GGA​AGA​ATG​GGA​GTT​GCT-3'; for Il-6, forward 5′-GAT​ACC​ACC​CAC​AAC​AGA​CCA-3′ and reverse 5′-CAG​AAT​TGC​CAT​TGC​ACA​ACT​C-3'; for Il-1β, forward 5′-GCT​TTC​GAC​AGT​GAG​GAG​AAT-3′ and reverse 5′-GCT​GCT​GTG​AGA​TTT​GAA​GCT-3'; and for Tnf-α, forward 5′-GAT​CGG​TCC​CAA​CAA​GGA​G-3′ and reverse 5′-CTT​GTC​ACT​CGA​GTT​TTG​AGA-3'. Data for cytokine mRNA were normalized to Gapdh expression and analyzed using the 2^−ΔΔCT^ method. For human Alu DNA detection, qPCR was performed using primers specific for the human Alu element (forward: 5′-CAT​GGT​GAA​ACC​CCG​TCT​CTA-3'; reverse: 5′-GCC​TCA​GCC​TCC​CGA​GTA​G-3′) on 100 ng of genomic DNA per reaction, with the same rat Gapdh primers co-amplified as a DNA loading control to verify equal genomic DNA input across samples.

### ELISA

Serum IL-2 and IFN-γ, as well as tissue IL-6, IL-1β, and TNF-α, were measured using commercial enzyme-linked immunosorbent assay (ELISA) kits (IL-2: F15820, WestTang; IFN-γ: F15730, WestTang; IL-6: F15870, WestTang; IL-1β: F15810, WestTang; TNF-α: F16960, WestTang) according to the manufacturer’s protocols. Serum samples were obtained by centrifugation at 1,500 × g for 15 min at 4 °C and stored at −80 °C until assay. For tissue cytokine measurement, vessel tissues were homogenized in RIPA lysis buffer containing protease inhibitors, and supernatants were collected after centrifugation at 12,000 × g for 20 min at 4 °C. Total protein concentrations in tissue lysates were determined using a BCA assay, and cytokine levels were normalized to total protein content (pg/mg protein). All samples were measured in duplicate, and cytokine concentrations were calculated based on standard curves generated with recombinant standards provided in the kits. Absorbance was read at 450 nm using a microplate reader.

### Statistical analysis

The experimental data were analyzed using GraphPad Prism software, and are presented as mean ± SD. All experiments were performed with n = 5 biological replicates per group. Differences between sham and model groups were analyzed by unpaired t-test. Differences among multiple groups were analyzed by one-way ANOVA. Changes in collagen proportion and nucleated cell count at 2, 3, and 4 weeks after tissue transplantation were analyzed by one-way ANOVA. Changes in mRNA levels in different treatment groups and at different time points were analyzed by two-way ANOVA.

## Results

### Fabricating the VWTs with iPSCs

Figure 2A illustrates the schematic diagram of the key stages in the VWTs culture process. Human iPSCs that were maintained on Matrigel-coated culture dishes exhibited island-like or nest-like colonies (Figure 2B- Day 0). After being transferred to ultra-low attachment surface plates for 2 days, iPSCs spontaneously formed spherical embryoid bodies (EBs) (Figure 2B–Day 4). Following this, the EBs were sandwiched between two layers of Matrigel, which transformed the three-dimensional EBs into a sheet-like form (Figure 2B- Week 4). Over several weeks of cultivation, the size of VWTs increased, with their diameters reaching between 3 and 5 mm (Figure 2B- Week 8). The VWTs adopted a flat-sheet morphology, which allows them to be coiled for insertion into the aneurysm sac using a standard 5 F catheter, a tool commonly used in clinical aneurysm sac filling procedures.

**FIGURE 2:**
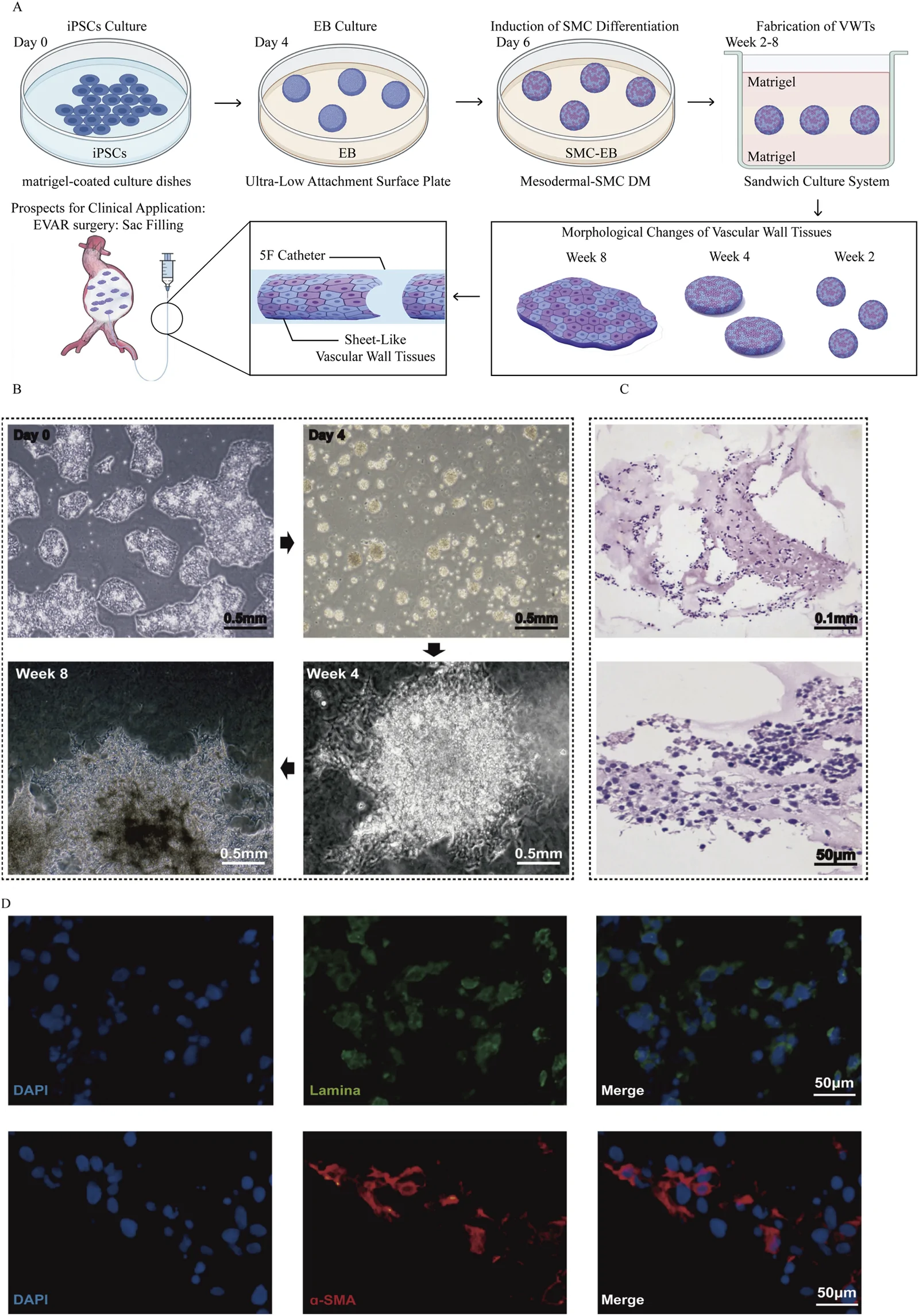
iPSCs culture and fabrication of vascular wall-like tissues. **(A)** Schematic diagram of the key stages in the VWTs culture process; **(B)** Human iPSCs were maintained on Matrigel-coated culture dishes to maintain stem cell properties, forming island-like or nest-like colonies (Day 0). The cells were then cultured on ultra-low attachment surface plates, where they spontaneously formed spherical embryoid bodies. These embryoid bodies were cultured in EB-mesodermal differentiation medium for 2 additional days to induce differentiation into mesodermal cells (Day 4). Subsequently, PDGF and TGF-beta were added to induce mesodermal cell differentiation into SMC-lineage-associated cells. The resulting cells were transplanted between Matrigels and cultured (Week 4). Cells exhibited continuous outward growth in a 12-well plate, with tissue size significantly increased by Week 8; **(C)** Hematoxylin and eosin staining showed the structure of the vascular wall-like tissues; **(D)** The differentiated cells were characterized using immunofluorescence staining for smooth muscle alpha-actin (α-SMA).

The internal tissue structure of the VWTs consisted of both cellular tissues and extracellular matrix (Figure 2C). Immunofluorescence staining was employed to identify α-SMA, a commonly used marker associated with smooth muscle lineage. The percentage of α-SMA^+^ cells was 71.76% ± 4.34% (Figure 2D). These results indicate that α-SMA^+^ (smooth muscle–lineage) cells are the predominant constituent of the VWTs. Additionally, human-specific lamin A expression was observed in VWT cells, confirming their human origin and enabling distinction from host rat cells in subsequent *in vivo* experiments.

### Establishment and characterization of a novel model of aneurysm with endoleak

To investigate the biological effects of VWTs on the aneurysm sac, we established a rat aneurysm-type II endoleak model and validated the model’s effectiveness from two aspects: the characteristics of the aneurysm vessel wall and the hemodynamic features of the type II endoleak (Figure 3A). After surgery, the maximum diameters of the aneurysms were measured *in vivo* and were significantly larger compared to the sham group (1.11 ± 0.08 mm vs. 0.62 ± 0.04 mm; P < 0.0001) (Figure 3B). Four weeks after surgery, the maximum diameter of the aneurysms *in vivo* decreased slightly to 1.01 ± 0.07 mm (Figure 3B). Aneurysm tissues were collected 4 weeks after surgery, and histological analyses of the aneurysm cross-sections were depicted in Figure 3C. The Model group exhibited a significantly larger diameter compared to the sham group (0.85 ± 0.12 mm vs. 0.62 ± 0.03 mm; P < 0.05) (Figure 3D). The degree of aortic wall structural damage was assessed based on the grade of elastin fragmentation (Figures 3E,F). The Model group displayed a significantly higher degree of elastic fragmentation compared to the sham group (3.67 ± 0.58 vs. 1.33 ± 0.58; P < 0.01). These results provided compelling evidence for the successful creation of the aneurysm model.

**FIGURE 3:**
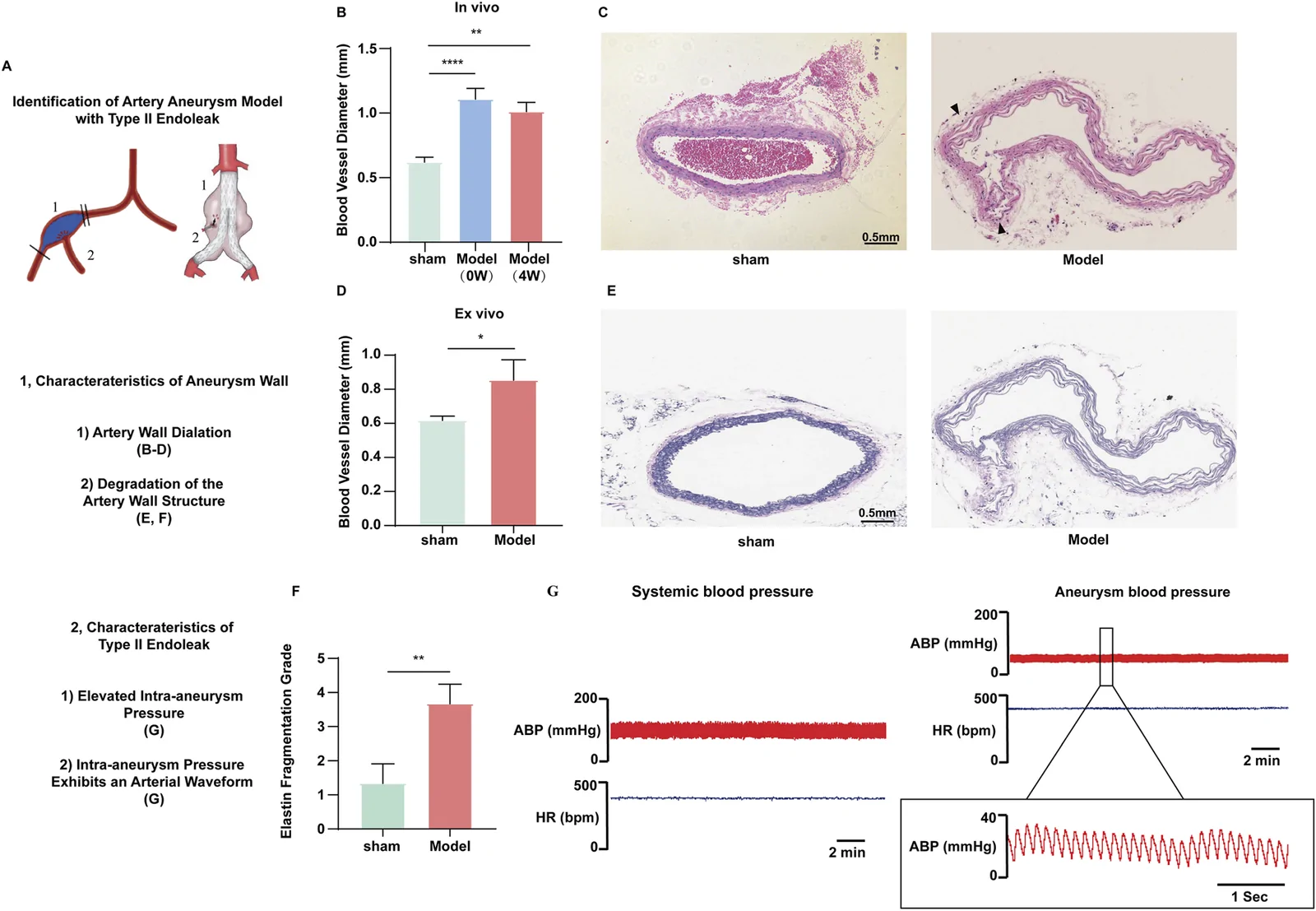
Validation of the model of aneurysm with endoleak. **(A)** Characteristics of arterial aneurysm-Type II endoleak model; **(B)** Maximum diameter of the *in vivo* aneurysm; **(C)** Representative Hematoxylin and Eosin staining of the vessel cross-sections in model group and sham group (arrows: elastin fragmentation); **(D)** The blood vessel diameter in model group and sham group; **(E)** Representative EVG staining of the vessel cross-sections in model group and sham group; **(F)** The elastin fragmentation grade in model group and sham group; **(G)** Representative original recordings of arterial blood pressure (ABP) and heart rate (HR) of systemic circulation and aneurysm sac. Values are mean ± SD; n = 5 in each group. *P < 0.05, **P < 0.01.

Retrograde arterial blood flow is a key factor in the occurrence of endoleaks. After completing the model construction, we confirmed the presence of retrograde blood flow and endoleak by continuously drawing arterial blood from within the aneurysm sac (Figure 3G). Intra-aneurysm blood pressure serves as a critical indicator for assessing the extent of internal leakage. The results indicated that the mean arterial pressure (MAP) in rats was 102.30 ± 6.50 mmHg, whereas the intra-aneurysm MAP measured 19.20 ± 5.10 mmHg (Figure 3G; Table 1), representing approximately 18.80% of the systemic MAP. As illustrated in Figure 3G, the blood pressure waveform within the aneurysm sac exhibited characteristic arterial pulsations. These findings substantiate the presence of internal leakage in the aneurysm model.

**TABLE 1:** The systemic blood pressure and intra-aneurysm blood pressure of rats.

Systemic MAP (mmHg)	Aneurysm MAP (mmHg)	Aneurysm SBP (mmHg)	Aneurysm DBP (mmHg)	Aneurysm pulse pressure (mmHg)
102.30 ± 6.50	19.20 ± 5.10	38.90 ± 12.90	10.00 ± 6.80	28.40 ± 8.90

Data are presented as mean ± SD (n = 5 per group). MAP, mean arterial pressure; SBP, systolic blood pressure; DBP, diastolic blood pressure.

Effect of VWTs transplantation on aneurysm sac embolization and tissue regeneration Firstly, to assess CsA immunosuppression and VWT effects, serum IL-2 and IFN-γ were measured using ELISA. Relative to vehicle group, CsA treatment reduced IL-2 and IFN-γ to 0.32 ± 0.07 and 0.48 ± 0.09 fold, respectively; CsA + VWTs yielded comparable values (0.34 ± 0.06 and 0.45 ± 0.08 fold). No significant differences existed between the two treatment groups, indicating that VWT engraftment did not alter CsA-mediated immune suppression.

After the transplantation of VWTs into the aneurysm sac, we continuously monitored the sac’s filling status and the growth of intra-sac tissue. The embolization and sac filling effects of VWTs transplantation were analyzed through the histological images of the aneurysm tissue at 2, 3, and 4 weeks after VWTs transplantation, with the blank Matrigel used for tissue culture serving as the control group (Figure 4A). The total embolization area proportion was defined as the ratio of all solid materials within the aneurysm sac, including both solid filling and blood clot filling (Figure 4B), while the proportion of solid embolization area was defined as the ratio of the area filled with tissue blocks and Matrigel within the aneurysm sac (Figure 4C). At 2 weeks post-surgery, the aneurysm sac filled with VWTs exhibited solid and total embolization area proportions of 45.17% ± 5.81% and 46.37% ± 4.25%, respectively (Figures 4A–C). The solid filling area was primarily composed of Matrigel and nucleated cells. Over time, both the solid and total embolization areas continued to increase. By 3 weeks after VWTs transplantation, these proportions reached 64.77% ± 18.01% and 76.77% ± 23.64%, respectively. Notably, at the fourth week, the solid and total embolization areas in the VWTs transplantation group were significantly higher than those in the Matrigel group, reaching 85.73% ± 11.44% and 98.83% ± 0.42%, respectively (Figures 4A–C). In contrast to the second week, the solid filling area within the sac was predominantly composed of cellular components rather than Matrigel, indicating the significant regenerative impact of the VWTs.

**FIGURE 4:**
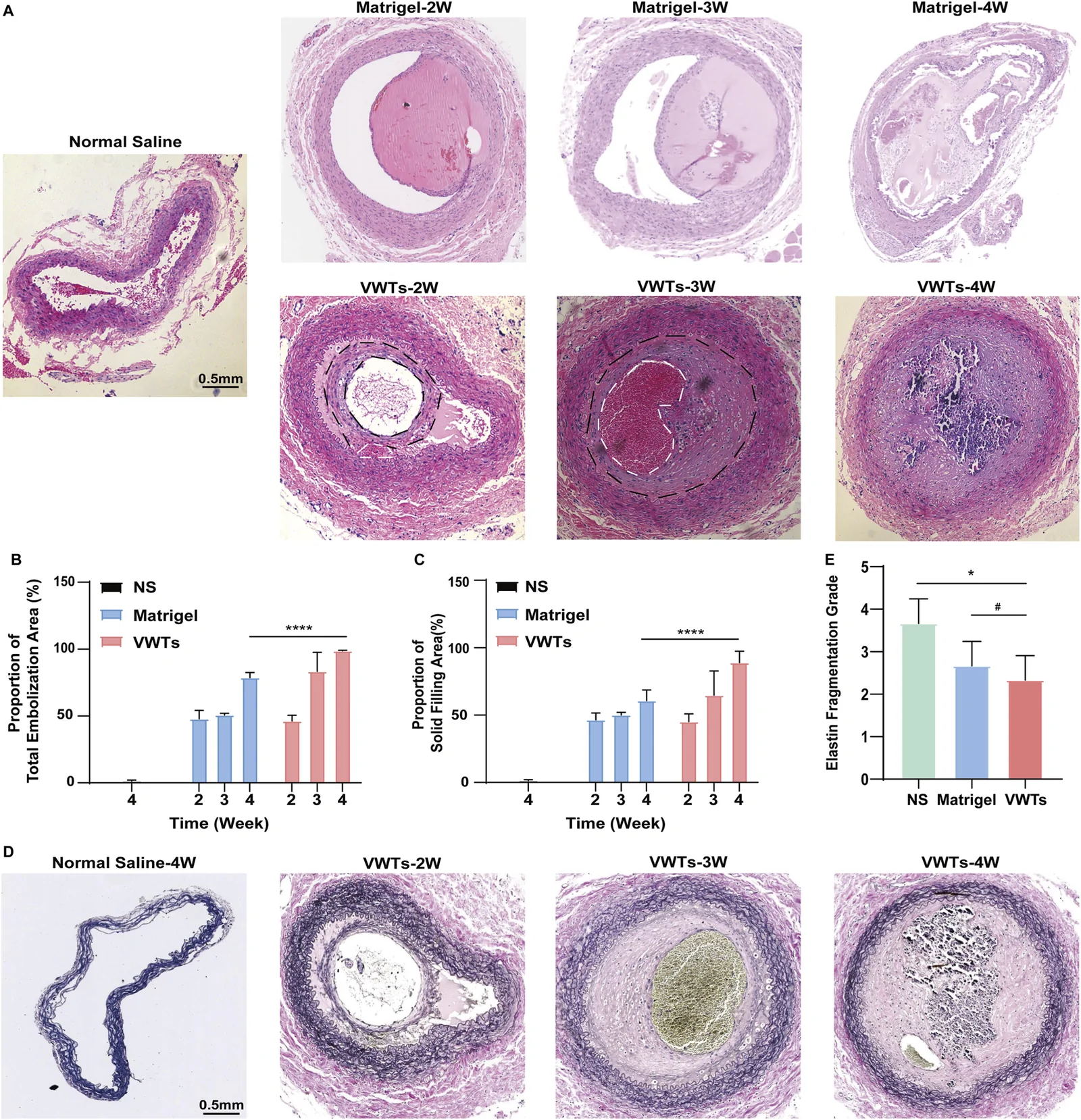
Effects of vascular wall-like tissues on aneurysm sac embolization and tissue regeneration. **(A)** Representative Hematoxylin and Eosin staining of vessel cross-sections after sac filling with normal saline (NS), Matrigel, or VWTs (black dashed box: solid filling area, white dashed box: blood clot filling); **(B,C)** Proportion of total embolization area and solid filling area in the aneurysm sac in aneurysm model after sac filling with normal saline (NS), Matrigel, or VWTs; **(D)** Representative EVG staining of vessel cross-sections; **(E)** The elastin fragmentation grade in aneurysm model, 4 weeks after normal saline injection or vascular wall-like tissues transplantation. Values are mean ± SD; n = 5 in each group. *P < 0.05, **P < 0.01, ***P < 0.001, ****P < 0.0001, #P > 0.05.

Furthermore, embolization with VWTs provided protection against further damage to the aneurysm. As demonstrated in Figure 4D, the VWTs treatment group exhibited significantly reduced elastic fragmentation compared to the control group 4 weeks after treatment (2.33 ± 0.58 vs. 3.67 ± 0.58; P < 0.05) (Figure 4E).

### The aneurysm sac remodeling effect mediated by VWTs

Figure 5A illustrates the schematic diagram of aneurysm sac structural reconstruction and the integration of transplanted and host-derived cells following VWT transplantation. Firstly, we examined the growth of the transplanted VWTs within the aneurysm sac. The VWTs tissue was identified through the determination of cellular origin. The VWTs were constructed from human iPSCs. To determine whether the regenerated tissue originated from the transplanted VWTs, human graft-derived cells were identified using a human-specific anti-lamin A antibody (red fluorescence, Figure 5B). During the first 3 weeks after VWTs transplantation, the proportion of cells originating from the transplanted tissue (lamin A-positive cells) increased with time (2 W: 50.82% ± 4.724% vs. 3 W: 65.41% ± 5.78%; P < 0.05), while the proportion remained stable in the fourth week after transplantation (64.40% ± 1.78%) (Figure 5C). These cells were primarily concentrated in the center of the aneurysmal sac, while newly generated cells recruited by the host organism were predominantly located around the sac. These results indicate that the transplanted VWTs and host tissue cells jointly participate in the process of aneurysm sac remodeling. Within 4 weeks after transplantation, VWT tissue was the predominant cellular source within the aneurysm sac. However, between 3 and 4 weeks post-transplantation, the efficiency of autologous cell recruitment and engraftment surpassed the growth of VWTs.

**FIGURE 5:**
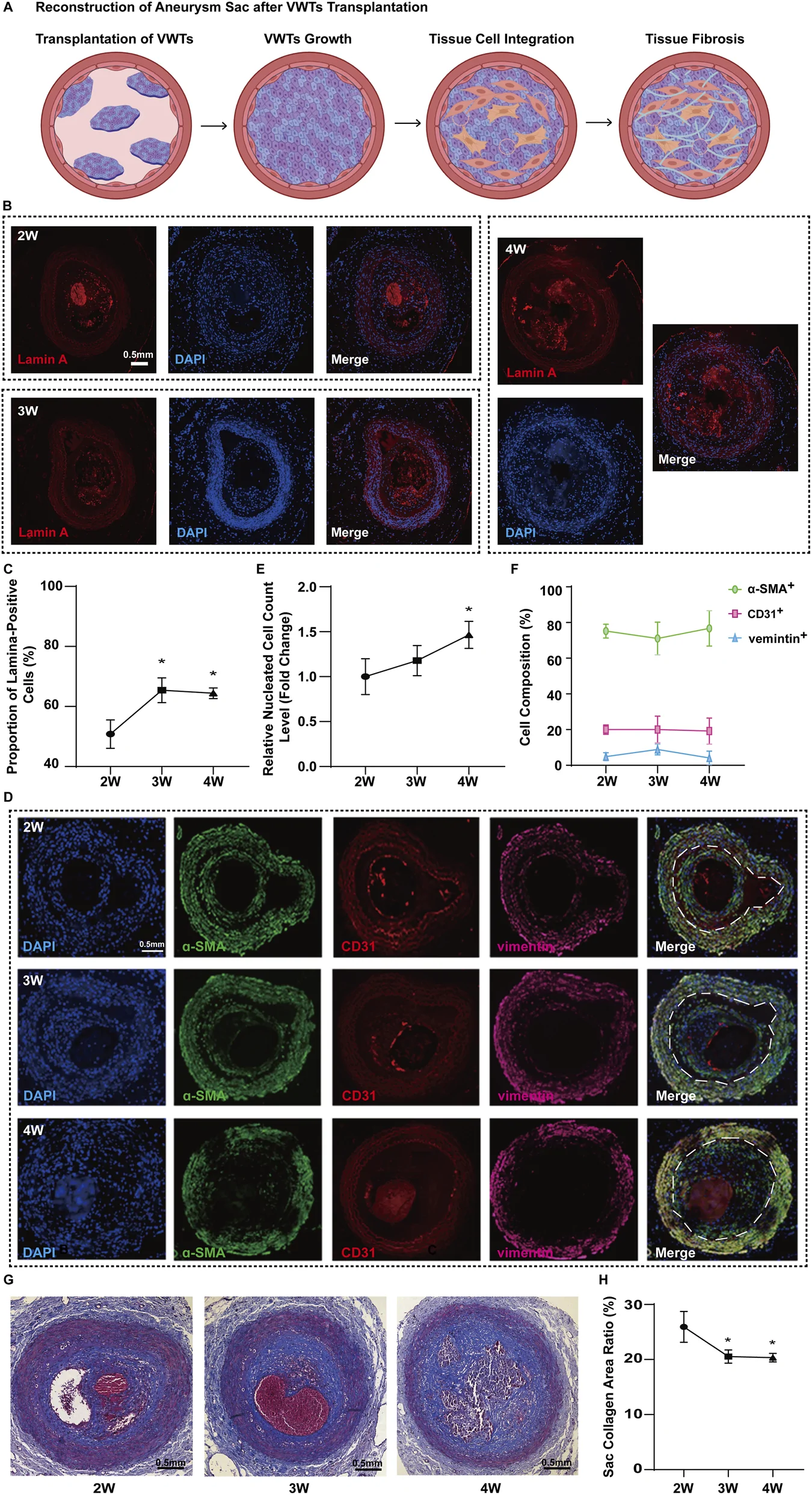
The aneurysm sac remodeling effect mediated by VWTs. **(A)** Schematic diagram illustrating aneurysm sac reconstruction and integration of transplanted and host-derived cells following VWT transplantation; **(B)** Representative immunofluorescence images of aneurysm cross-sections acquired 2, 3 and 4 weeks after VWT transplantation, showing the graft-derived human cells labelled with anti-lamin A antibody (red). **(C)** Proportion of lamin A-positive cells in the aneurysm sac from aneurysm models with vascular wall-like tissues transplantation after 2, 3, and 4 weeks; **(D)** Representative fluorescence images of aneurysm sac in the model group with vascular wall-like tissues transplantation; dashed lines indicate the boundary between the original vascular wall and the reconstructed sac; **(E,F)** Relative nucleated cell count level and cell type composition in the aneurysm sac in the model group with vascular wall-like tissues transplantation after 2, 3, and 4 weeks; **(G)** Representative Masson’s trichrome staining of vessel cross-sections; **(H)** Proportion of fibrotic area within the sac from aneurysm models with vascular wall-like tissues transplantation after 2, 3, and 4 weeks. Values are mean ± SD; n = 5 in each group. *P < 0.05 vs. 2 W.

We conducted further investigations into the histological characteristics of regenerative tissue. Cellular components within the aneurysm sac were analyzed through immunofluorescence (Figure 5D). The content of nucleated cells within the aneurysm sac increased with time, exhibiting a fold change of 1.18 ± 0.17 and 1.68 ± 0.15 (P < 0.05) in the 3-week and 4-week groups, respectively, when compared to the 2-week group post-treatment (Figure 5E). We also investigated the distribution and proportion of three marker-defined cell populations within the regenerative tissue. As indicated in Figure 5F, the proportions of these 3 cell populations remained relatively constant during the regeneration process. α-SMA^+^ cells accounted for the largest proportion in the regenerative tissue (2 W: 79.30% ± 6.62%, 3 W: 74.40% ± 14.13%, 4 W: 76.90% ± 10.18%), followed by CD31^+^ cells (2 W: 20.00% ± 2.65%, 3 W: 20.03% ± 7.48%, 4 W: 19.17% ± 7.33%) and vimentin^+^ cells (2 W: 4.83% ± 2.20%, 3 W: 8.90% ± 3.01%, 4 W: 4.13% ± 3.79%).

Figure 5G illustrates the changes in collagen content within the aneurysm sac following VWTs transplantation. Two weeks after transplantation, the proportion of collagen in the sac reached 25.93% ± 2.793%. However, over time, there was a decrease in collagen content, with ratios of 20.53% ± 1.20% and 20.33% ± 0.78% observed after three and 4 weeks, respectively (Figure 5H).

### Preliminary Short-Term *In Vivo* Safety Assessment

We next conducted a preliminary short-term *in vivo* safety assessment of VWT transplantation. Examination of aneurysm sac tissue sections at 2–4 weeks post-transplantation revealed no evidence of teratoma formation, a finding that would have been characterized by the presence of all three germ layers (Figure 6A). We further performed the preliminary short-term *in vivo* safety assessment of VWTs by assessing their biodistribution. At 4 weeks following transplantation of VWTs into the aneurysm sac, we harvested aneurysm sac tissue and six organs (brain, lung, heart, liver, kidney, and spleen) to search for migration of human-origin cells from the VWTs grafts. Human-specific Alu DNA was detectable in the aneurysm sac but was not detectable in any of the six examined distant organs, including the brain, lung, heart, liver, kidney, and spleen (Figure 6B), indicating no detectable dissemination of graft-derived human cells to the examined distant organs within the 4-week observation period. We further evaluated the inflammatory response levels following the transplantation of VWTs. Peripheral vascular wall tissues from the aneurysms in the normal saline (NS) filling group, Matrigel filling group, and the VWTs filling group were collected at 2, 3, and 4 weeks post-surgery, and the levels of inflammatory cytokines were assessed (Figures 6C–E). We selected the peripheral vascular wall tissue because it represents the adjacent native vascular tissue surrounding the treated aneurysm sac and can reflect whether VWT transplantation imposes an additional inflammatory burden on the surrounding vessel. In contrast, measurements from sac contents alone may be confounded by injected materials, transplanted cells, and remodeling tissue within the sac. The findings revealed that IL-6, IL-1β, and TNF-α mRNA levels in the peripheral vascular wall tissues of the aneurysms were significantly increased after surgery, with the highest levels observed at 2 weeks post-surgery. Most importantly, the levels of IL-6, IL-1β, and TNF-α mRNA in the VWTs group were consistently not higher than those in the NS group and Matrigel group (Figures 6C–E). This suggested that the use of VWTs for filling did not induce a strong inflammatory response in the adjacent native vascular tissue, although the inflammatory microenvironment within the sac itself requires further detailed evaluation.

**FIGURE 6:**
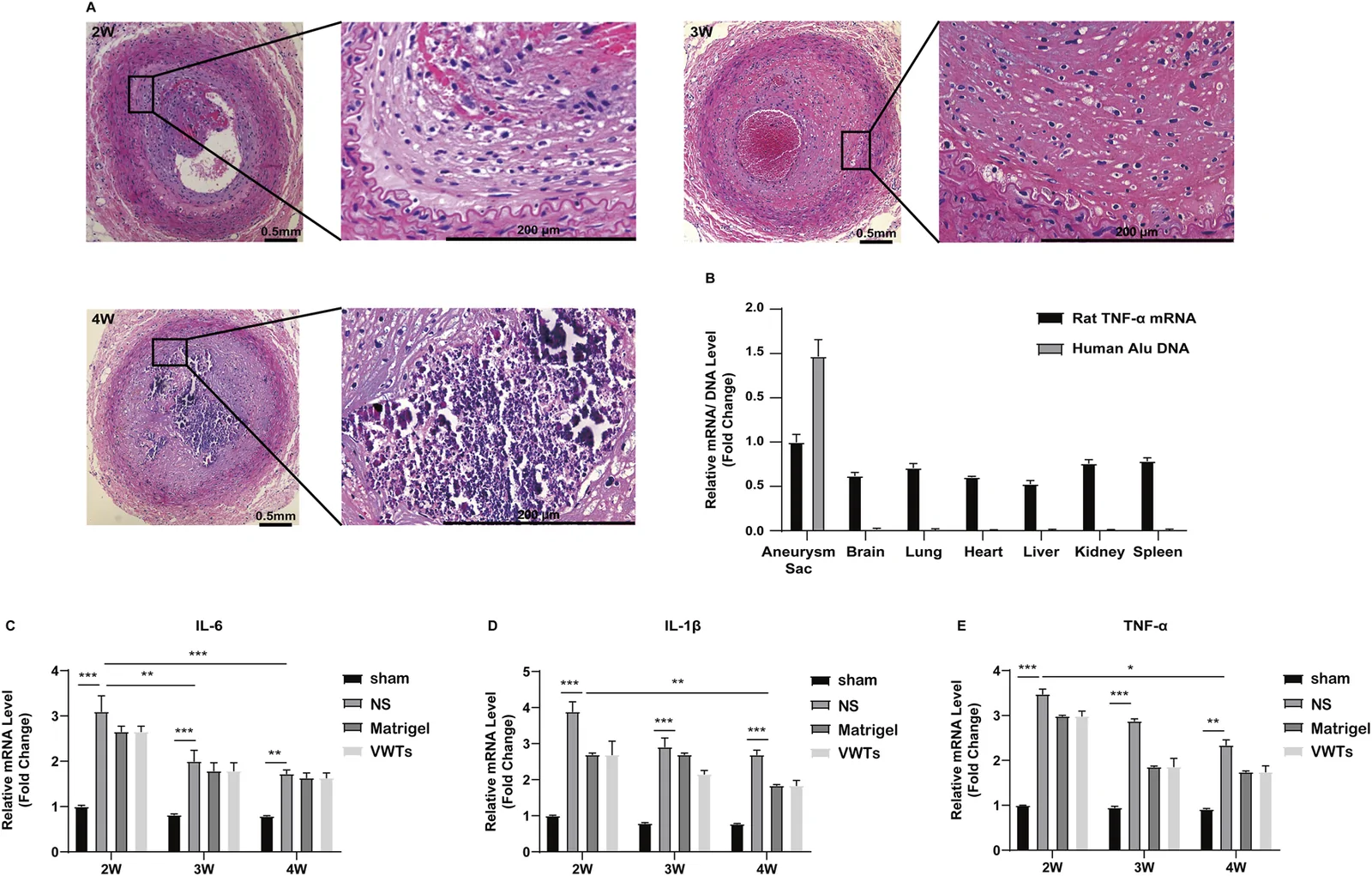
Preliminary Short-Term *In Vivo* Safety Assessment. **(A)** Representative Hematoxylin and Eosin staining of the vessel cross-sections, 2, 3, and 4 weeks after VWT transplantation; **(B)** Relative mRNA levels of rat TNF-α and relative DNA levels of human Alu in the aneurysm sac, brain, lung, heart, liver, kidney, and spleen of rats, 4 weeks after vascular wall-like tissues transplantation; **(C–E)** Relative mRNA levels of IL-6, IL-1β, and TNF-α in the peripheral vascular wall tissues of the aneurysms. Values are mean ± SD; n = 5 in each group. *P < 0.05, **P < 0.01, ***P < 0.001.

## Discussion

The present study aimed to explore the efficacy of vascular wall-like tissues in aneurysm sac embolization and tissue regeneration, and their potential application in addressing endoleaks after EVAR. In summary, we conducted *in vitro* cultivation and induction of iPSC clusters in Matrigel to fabricate the VWTs. Filling the surgically created aneurysm with endoleak using VWTs produced favorable effects on aneurysm sac embolization and tissue regeneration.

We established a rat aneurysm-endoleak model as a complementary small-animal platform for preliminary evaluation of bioactive sac-filling agents, while recognizing that large-animal models remain essential for translational validation. The commonly used aneurysm models involve creating a weakness in arterial segments by transplanting a patch, including antigen-processed bovine jugular vein or artificial patch, into the abdominal aorta (Diaz et al., 2004). These surgically created aneurysm walls lack vascular cells and structures, failing to provide necessary oxygen and nutrients for the implanted biomaterials and making it difficult to observe critical processes of thrombus organization and tissue regeneration within the aneurysm sac (Diaz et al., 2004; Pavcnik et al., 2003; Cornelissen et al., 2012). In order to address this issue, in our model, we performed mechanical dilation of the iliac artery and external iliac artery to simulate the underlying pathological condition of aortic aneurysm, including injured arterial wall structure, which could then induce physiological or pathophysiological responses such as inflammation and neovascularization (Kaneko et al., 2011; Suzuki et al., 2020). Morphological changes (enlarged diameter and disrupted wall structure) confirmed successful aneurysm modeling, while patency of the internal iliac artery recapitulated retrograde collateral perfusion, with intra-sac pressure (approximately 19% of systemic pressure) and arterial sinusoidal waveform resembling clinical type II endoleaks (Dias et al., 2007).

Nevertheless, substantial interspecies differences must be acknowledged. Vascular geometry, biomechanics, and hemodynamics vary markedly across species and can influence the distribution, retention, and remodeling of embolic materials. In particular, the rat iliac artery is approximately 40- to 60-fold smaller than a human AAA, with correspondingly different flow rates, pressure gradients, and wall shear stress, which together limit direct extrapolation to the clinical setting (Isselbacher et al., 2022; Schmitz-Rixen et al., 2020; Dias et al., 2007). Large-animal models (e.g., porcine, canine, or ovine) more closely approximate human vessel caliber, flow patterns, and wall architecture, providing a more realistic post-EVAR environment, albeit at greater cost and over longer timelines. We therefore position the present rat model as an initial feasibility platform for validating the fundamental biological effects of VWTs, and emphasize that confirmation in clinically relevant large-animal models will be essential before clinical translation can be considered.

An important aspect of this study is the design of the VWTs. We selected iPSCs because of their broad availability, strong proliferative capacity, and multilineage differentiation potential (Aboul-Soud et al., 2021; Thanaskody et al., 2022). We are nonetheless aware of the translational challenges of iPSC-derived products, including residual undifferentiated cells, teratoma risk, and batch-to-batch heterogeneity, which demand rigorous quality control and longer-term safety evaluation before clinical use (Thanaskody et al., 2022). To begin addressing these concerns, we performed a preliminary short-term *in vivo* safety assessment in the present study. Our findings revealed no evidence of teratoma formation, no detectable dissemination of graft-derived cells to distant organs, and no exacerbated inflammatory responses in the adjacent native vascular tissue compared with controls. However, these observations are limited to a 4-week follow-up period. Importantly, the potential presence of residual undifferentiated cells and the associated long-term tumorigenic risk remain unresolved issues that require extended observation and further evaluation in future studies. Rather than attempting to reconstruct a fully stratified vascular wall *in vitro*, we deliberately engineered the VWTs as α-SMA^+^ cell-rich sheets. This choice was guided by the pathophysiology of the post-EVAR aneurysm sac, which is typically ischemic, hypoxic, and nutrient-poor—conditions under which a complex, multi-layered tissue would be unlikely to survive (Bashur et al., 2013). Although α-SMA staining alone does not establish mature, contractile smooth muscle identity, SMC-lineage–associated cells are known to drive extracellular matrix production and vascular wall remodeling (Nakamura et al., 2023; Nevzati et al., 2020; Chen et al., 2020; Frösen et al., 2019). Consistent with this rationale, α-SMA^+^ cells remained the predominant population within the regenerated tissue, supporting the view that an α-SMA^+^ cell-rich sheet can act as a viable seed construct that promotes provisional matrix deposition, host-cell recruitment, and gradual sac remodeling within this challenging microenvironment.

A key rationale for our approach is the use of three-dimensional cell clusters rather than dissociated cell suspensions. Engineered cell clusters provide higher cell density, greater transplantation efficiency, and more active intercellular communication, potentially leading to improved viability and enhanced angiogenesis (Takeuchi et al., 2016; Komae et al., 2017). These advantages are particularly relevant within an endoleak-containing aneurysm sac, where the cluster format offers greater mechanical stability and better preserves the transplanted cells. Consistent with this, graft-derived (lamin A^+^) cells persisted within the sac for at least 4 weeks after transplantation and accounted for more than 50% of the nucleated cells, highlighting the feasibility of tissue transplantation for aneurysm sac embolization.

We next examined how VWTs mediate tissue regeneration and sac remodeling. First, the transplanted VWTs progressively integrated with the aneurysm wall. At early time points, the cellular components of the VWTs were arranged mainly around the residual sac, with Matrigel interposed between these components and the arterial wall; by three to four weeks, this Matrigel was largely replaced by cellular components. Immunofluorescence further revealed a near-continuous layer of CD31^+^ endothelial cells along the inner surface of the residual sac. Together, these findings suggest that the VWTs integrated with the native arterial wall to form a composite-like vascular wall structure. Second, host biological processes also contributed substantially to sac remodeling: graft-derived cells were predominantly located in the center of the sac, whereas host-derived cells resided mainly in its periphery. This spatial pattern underscores the active role of host tissue and endogenous repair in aneurysm sac regeneration and supports the value of sac-filling agents that actively engage host-mediated repair and remodeling. Finally, the decrease in sac collagen from 25.9% at 2 weeks to approximately 20% at 3–4 weeks post-transplantation likely reflects a normal transition from acute repair to mature remodeling. Two biologically plausible explanations can be hypothesized: first, the early collagen signal partially originates from the Matrigel carrier, which is gradually degraded by the host (Kleinman and Martin, 2005); and second, the subsequent reduction could involve active matrix remodeling, potentially mediated by matrix metalloproteinases or other enzymatic activities, during which excess provisional matrix is trimmed toward a more organized architecture (Chen et al., 2020). Collectively, the observed decrease in collagen content may be consistent with ongoing matrix remodeling rather than therapeutic failure. However, because Matrigel degradation, collagen turnover, and matrix metalloproteinase activity were not directly assessed, the underlying mechanisms remain speculative and require further investigation.

Several limitations of the present study should be noted. First, as discussed above, our rat iliac-artery model differs substantially from human AAA in scale, biomechanics, and hemodynamics; accordingly, this work is intended as a biological proof-of-concept—testing whether VWTs can survive, engraft, and drive tissue remodeling within a pressurized sac—rather than a demonstration of direct clinical feasibility, and confirmation in large-animal models remains necessary. Second, the VWTs culture takes 1–2 months, which may cause some inconvenience in clinical usage. Therefore, in clinical practice, for non-ruptured AAAs, patient tissue can be collected in advance, and VWTs can be cultured and implanted into the aneurysm sac during elective EVAR surgery. More importantly, based on this study, we consider using gene editing technologies like CRISPR-Cas9 to eliminate key immune recognition molecules from the cell surface, such as MHC class I and II molecules, or to introduce immunomodulatory factors like PD-L1 to engineer stem cells and vascular wall-like tissues. This may enable off-the-shelf production in the future; however, substantial safety and regulatory validation would be required before clinical application.

This study is rooted in clinical practice and aims to address a practical clinical problem (Wu et al., 2026). The prevention and treatment of type II endoleaks remain challenging. Our center has been at the forefront of using fibrin sealant for intra-aneurysmal coagulation and sac filling, achieving satisfactory short- and mid-term outcomes (Lu et al., 2010; Chen et al., 2024). However, mid-to-long-term efficacy in preventing type II endoleaks has not improved substantially, likely because intra-aneurysmal clots undergo fragmentation, absorption, and displacement, leading to recurrent sac reperfusion. In addition, artificial embolic agents such as coils and chemical glues carry risks of compression, deformation, and displacement that may compromise their long-term efficacy. Against this background, our study introduces a new concept for post-EVAR management: achieving durable sac occlusion through biological remodeling rather than purely mechanical filling. The VWTs we designed have the notable advantage of promoting sac remodeling and host-cell integration, and occlusion mediated by the body’s own tissue may be more stable and durable, offering a promising strategy for endoleaks arising from a residual aneurysm sac after EVAR. Importantly, the two approaches are complementary: intraluminal coagulation strategies such as fibrin sealant could be combined with VWTs to secure early embolization while driving biological sac remodeling, thereby promoting long-term, stable sac consolidation.

In summary, this study successfully established a novel rat aneurysm model that recapitulates key features of type II endoleak, providing a practical small-animal platform for evaluating bioactive sac-filling strategies. Using this model, we further demonstrate that VWTs—three-dimensional cultured cell clusters—can promote tissue regeneration and remodeling of the aneurysm sac, with potential for durable, stable embolization after EVAR. These results highlight the advantage of three-dimensional cell clusters over dissociated cell suspensions for aneurysm sac embolization. More broadly, our work supports a shift toward sac-filling strategies that actively engage the host’s intrinsic repair and remodeling processes, which we believe represents a promising direction for future research on post-EVAR endoleak management.

## Data Availability

The original contributions presented in the study are included in the article/supplementary material, further inquiries can be directed to the corresponding authors.
